# Impulse control disorder in Parkinson’s disease: the influence of dopamine agonists

**DOI:** 10.1192/j.eurpsy.2026.11496

**Published:** 2026-07-31

**Authors:** G. F. Varandas Rodrigues, A. T. Pereira

**Affiliations:** Psychiatry and Mental Health, Unidade Local de Saúde do Alto Minho, Viana do Castelo, Portugal

## Abstract

**Introduction:**

Parkinson’s Disease (PD) is a chronic neurodegenerative pathology, characterized by nigrostriatal dopaminergic dysfunction and motor manifestations. Dopamine agonists (DA) are one of the pillars of treatment. However, the stimulation of dopamine receptors in the mesolimbic pathways has been associated with the appearance of neuropsychiatric adverse effects, namely impulse control disorders (ICD), including pathological gambling, hypersexuality, impulsive shopping and binge-eating. These clinical manifestations have a significant clinical impact, jeopardizing patients’ functionality and quality of life.

**Objectives:**

Review the literature regarding the scientific evidence on the association between the use of DA and the development of ICD in patients with PD.

**Methods:**

Non-systematic review based on a search for scientific articles, between 2010 and 2024, in Pubmed using the MeSH terms: ‘dopaminergic agonists’, ‘impulse control disorder’, ‘Parkinson’s disease’.

**Results:**

The analysis published in 2022, based on data collected between 1968 and 2020, showed a high incidence of ICD in patients medicated with dopamine agonists. Pramipexole and ropinirole showed a clear association with pathological gambling, hypersexuality and impulsive buying behavior.

A prospective study by Weintraub found that 17.1 percent of patients exposed to DA developed at least one form of ICD. It should be noted that behavioral symptoms showed a dose-dependent relationship, and in many cases regressed after the agonist was reduced or discontinued. The risk of ICD associated with ropinirole increases proportionally with the plasma concentration of the drug, while with pramipexole, the risk remains high even at lower serum levels, suggesting an intrinsic affinity of this compound for D3 receptors in the limbic regions.

On a neurobiological level, in patients with ICD, a significant reduction in the availability of D3 receptors in the ventral striatum and an increase in the release of dopamine in this region in response to reward stimuli, such as risky behavior, were observed. This supports the hypothesis of limbic dopaminergic sensitization induced by chronic exposure to DA.

Several predisposing factors have been identified as potentiating the risk of developing ICD, such as early onset of PD, personal and family history of addictive behaviors, impulsive personality traits, and pre-existing psychiatric comorbidity.

**Conclusions:**

Scientific evidence shows a clear association between the use of dopamine agonists, particularly pramipexole and ropinirole, and the development of ICD in Parkinson’s patients. This relationship is mediated by neurobiological mechanisms involving D3 dopamine receptors and reward circuits. The presence of individual risk factors reinforces the importance of regular monitoring of risk behaviors, optimizing therapy and educating patients and carers in order to prevent and intervene effectively in these situations.

**Disclosure of Interest:**

None Declared

